# Clinical Examination of the Urine and Urinary Diagnosis

**Published:** 1901-05

**Authors:** 


Clinical Examination of the Urine and Urinary Diagnosis.—A clinical guide for the use of practitioners and students of medicine and surgery. By J. Bergen Ogden, M. D., Instructor in Chemistry, Harvard University Medical School; Assistant in Chemical Pathology, Boston City Hospital; Medical Chemist to the Carney Hospital; Visiting Chemist to the Long Island Hospital, Boston. Illustrated. 416 pages. Price, $3.00. Philadelphia: W. B. Saunders & Co. 1900.

This book departs a little from the usual work on the urine in that it takes up in detail the subject of urinary diagnosis and applies the information furnished by careful chemic and microscopic examinations. In other words, it presents in a clear and concise manner the chemistry of the urine and its relation to physiologic processes. The methods of investigation are simple and scientific.

Part I is devoted to the chemic and microscopic methods, and has scattered through it a sufficient number of illustrations to make clear the text.

Part II gives attention to diagnosis, and includes everything modern in connection with disturbances and diseases of the kidneys and urinary passages whether they be local or general, medical or surgical. The sale of this book should be large.

